# Alpha-Mangostin-Loaded Transferrin-Conjugated Lipid-Polymer Hybrid Nanoparticles: Development and Characterization for Tumor-Targeted Delivery

**DOI:** 10.1155/2022/9217268

**Published:** 2022-08-30

**Authors:** Intouch Sakpakdeejaroen, Patcharaporn Muanrit, Sumalee Panthong, Srisopa Ruangnoo

**Affiliations:** ^1^Department of Applied Thai Traditional Medicine, Faculty of Medicine, Thammasat University, Khlong Nueng 12120, Pathumthani, Thailand; ^2^Centre of Excellence, Applied Thai Traditional Medicine Research (CEATMR), Thammasat University, Khlong Nueng 12120, Pathumthani, Thailand

## Abstract

Alpha-mangostin, a natural xanthone mainly extracted from the pericarp of *Garcinia mangostana*, has been shown to have promising anticancer properties in many types of cancer. However, the therapeutic potential of *α*-mangostin has been limited so far due to its poor aqueous solubility and low oral bioavailability, which limited its biopharmaceutical applications. Furthermore, *α*-mangostin failed to specifically reach tumors at a therapeutic concentration due and rapid elimination *in vivo*. We hypothesized that this drawback could be overcome by loading the drug within a delivery system conjugated to transferrin (Tf), whose receptors are overexpressed on many cancer cells and would enhance the specific delivery of *α*-mangostin to cancer cells, thereby enhancing its therapeutic efficacy. The objectives of this study were therefore to prepare and characterize transferrin-conjugated lipid-polymer hybrid nanoparticles (LPHN) entrapping *α*-mangostin, as well as to evaluate their therapeutic efficacy *in vitro*. We successfully prepared *α*-mangostin loaded LPHN using a one-step nanoprecipitation method with high drug entrapment efficiency. The conjugation of Tf to the LPHN was achieved by using the thiol–maleimide “click” reaction, leading to an increase in the particle hydrodynamic size of Tf-LPHN compared to that of unconjugated (control) LPHN (Ctrl-LPHN). Both Tf-LPHN and Ctrl-LPHN were bearing negative surface charges. Tf-LPHN and Ctrl-LPHN exhibited a sustained release of *α*-mangostin at pH 7.4, following an initial burst release, unlike rapid release of drug solution. The entrapment of *α*-mangostin in the LPHN led to an increase in *α*-mangostin uptake by cancer cells, and thus improved its antiproliferative activity compared to that observed with the drug solution. In conclusion, *α*-mangostin entrapped in the Tf-LPHN is therefore a highly promising therapeutic system that should be further optimized as therapeutic tools for cancer treatment.

## 1. Introduction

Alpha-mangostin, a natural xanthone mainly extracted from the pericarp of *Garcinia mangostana* (commonly known as mangosteen), has a wide spectrum of pharmacological properties, for example, antiinflammation, antibacterial, antifungal, and antioxidant activities. Moreover, *α*-mangostin has recently gained considerable attention for its chemopreventive and therapeutic efficacies *in vitro* against many types of cancer, including lung, breast, liver, colon, prostate, cervical, and skin cancers [1]. It is well established that *α*-mangostin exerts its anticancer effect via multiple mechanisms such as enhancing the amount of reactive oxygen species, activation of focal adhesion kinase (FAK), apoptosis signal-regulating kinase 1 (ASK1)/p38, p53, poly-ADP ribose polymerase (PARP) and caspase-3, through suppression of human epidermal growth factor receptor 2 (HER2)/phosphatidylinositol-3-kinase (PI3K)/protein kinase B (Akt), and mitogen-activated protein kinase (MAPK)/extracellular signal-regulated kinases (ERK) as well as inhibition of folic acid synthesis (FAS) and ATP-binding cassette transporter G2 (ABCG2) [1–3]. This wide range of anticancer effects, therefore, makes *α*-mangostin a very promising therapeutic molecule. However, the therapeutic potential of *α*-mangostin has been limited so far due to its poor solubility in water (2.03 × 10^−4^ mg/L at 25°C), high lipophilicity (log *p* 7.71), and photolytic property [4, 5], which limited its biopharmaceutical applications. Furthermore, *α*-mangostin failed to specifically reach tumors at a therapeutic concentration due to its low oral bioavailability (less than 5%) and rapid elimination *in vivo*, with a short biological half-life of approximately 3.5 hours [6, 7].

This limitation could be solved by using a drug delivery system that is able to entrap a hydrophobic drug, improve its water solubility, prolong its blood circulation time, and sustain its release over a period of time. In addition, nanocarriers can enhance drug delivery to tumors via passive targeting through the enhanced permeability and retention (EPR) effect [8, 9]. Several types of nanocarriers have previously been investigated to minimize the drawbacks and improve the therapeutic potential of *α*-mangostin, such as polymeric nanoparticles, liposomes, solid lipid nanoparticles, and nanomicelles [1], but with limited antitumor efficacy so far. Besides, some side effects may occur due to the nonselective accumulation of nanocarriers in other organs (e.g., liver and spleen), which can be overcome by using active targeting [8, 10]. On the basis that iron is essential for cancer cell growth and can be effectively carried to tumors by transferrin (Tf), whose receptors are overexpressed on many cancer cells [11], we hypothesize that conjugating nanocarriers with transferrin would enhance the specific delivery of *α*-mangostin to cancer cells, thus improving its therapeutic efficacy.

Several nanocarriers have previously been investigated to enhance the therapeutic efficacy of *α*-mangostin including liposomes and polymeric nanoparticles [3]. Chen and colleagues developed Tf-liposome that was able to improve the delivery of *α*-mangostin across the blood-brain barrier *in vivo* [12]. Verma and colleagues formulated *α*-mangostin-loaded polymeric nanoparticles which enhanced its antiproliferative and apoptotic effects in pancreatic cancer stem cells and pancreatic cancer cell lines but had no effect on normal human pancreatic ductal epithelial cell lines [13]. Although liposomes and polymeric nanoparticles have many advantages such as biocompatibility, biodegradability, versatile drug loading, prolonged blood circulation time, sustained drug release, and protecting encapsulated drugs from physiological environments [1], some problems still exist. For example, liposomes are less stable due to hydrolysis and peroxidation of their fatty acids, and therefore have a limited half-life and high drug leakage during storage. Polymeric nanoparticles generally have some pitfalls, such as particle size variation and aggregation [8].

Therefore, in this study, we selected a new combination of drug delivery systems, lipid-polymer hybrid nanoparticles, which offered advantage characteristics of both lipid-based vesicles and polymeric nanoparticles, meanwhile, overcoming the drawbacks of these nanocarriers [14]. The biocompatible and biodegradable poly (lactide-co-glycolide) (PLGA) hydrophobic copolymer was used to form the polymeric core of nanoparticles, which are capable of entrapping *α*-mangostin and controlling its release. The outer layer of the PLGA core was surrounded by a monolayer of heterobifunctional PEGylation phospholipid and 1, 2-distearoyl-*sn*-glycero-3-phosphoethanolamine-N-(maleimide (polyethylene glycol)-2000) (DSPE-PEG2K-MAL), which provided a stealth effect, protected the entrapped drug from freely diffusing out, and facilitated surface modification with Tf.

The objectives of this study were therefore (1) to develop and characterize Tf-conjugated lipid-polymer hybrid nanoparticles entrapping *α*-mangostin and (2) to evaluate their cellular uptake and antiproliferative activity on cancer cells *in vitro*.

## 2. Materials and Methods

### 2.1. Chemicals and Reagents

Alpha-mangostin, Resomer® RG 503 H (acid-terminated poly (lactide-co-glycolide) (PLGA-COOH), lactide: glycolide 50 : 50, MW 24000–38000 Da, viscosity 0.32–0.44 dL/g), human holo-transferrin, 2-iminothiolane hydrochloride (Traut's reagent), 3-(4, 5-dimethylthiazol-2-yl)-2, 5-diphenyl-tetrazolium bromide (MTT), and all other chemicals that are not specifically mentioned below were purchased from Sigma Aldrich, Germany. DSPE-PEG2K-MAL was obtained from Jenkem Technology, USA. Acetone and dimethyl sulfoxide (DMSO) were purchased from RCI Labscan, Thailand. Dulbecco's Modified Eagle Medium (DMEM), minimal essential medium (MEM), Roswell Park Memorial Institute 1640 (RPMI-1640), fetal bovine serum (FBS), and penicillin-streptomycin were obtained from Gibco, USA.

### 2.2. Preparation of Transferrin-Conjugated Lipid–Polymer Hybrid Nanoparticles Entrapping *α*-Mangostin

The LPHN entrapping *α*-mangostin was prepared using the one-step nanoprecipitation method. Briefly, DSPE-PEG2K-MAL (5 mg) was shaken with deionized water (5 mL) at 65°C for 1 hour. A mixture of *α*-mangostin (4 mg) and PLGA-COOH (25 mg) was dissolved in acetone (2.5 mL). It was subsequently added dropwise into the lipid part under moderate stirring (700 rpm). The product was stirred overnight at 25°C under a chemical fume hood to remove all acetone.

In order to conjugate Tf to LPHN, a cross-linking Traut's reagent was used to produce a thiolated Tf which can interact with the thiol-reactive maleimide group of DSPE-PEG2K-MAL. To do so, Tf (1 mL, 10 mg/mL in 50 mM sodium phosphate buffer, 150 mM sodium chloride, pH 8.0) was reacted with a 10-fold molar excess of Traut's reagent (85 *µ*L, 2 mg/mL in deionized water) under continuous stirring at 25°C for 2 hours. The thiolated Tf was then separated from unreacted Traut's reagent using Amicon® ultra-4 centrifugal filter units (molecular weight cut-off of 3 kDa) by centrifugation at 5,000 rpm for 30 min at 25°C. The freshly synthesized thiolated Tf was immediately conjugated to the LPHN under continuous stirring at 25°C for 2 hours.

The resulting Tf-LPHN and Ctrl-LPHN were purified to remove unentrapped *α*-mangostin and/or unreacted Tf using Amicon® ultra-15 centrifugal filter units (molecular weight cut-off of 100 kDa) by centrifugation at 5,000 rpm for 20 min at 25°C. They were rinsed with deionized water (2 mL) before being resuspended in deionized water (1 mL). For empty nanoparticles (blank LPHN), they were prepared and conjugated with Tf in the same manner as Tf-LPHN but without *α*-mangostin. All nanoparticles were stored at 4°C for further experiments.

### 2.3. Characterization of Transferrin-Conjugated Lipid–Polymer Hybrid Nanoparticles Entrapping *α*-Mangostin

#### 2.3.1. Particle Morphology

The morphology of the LPHN was visualized by transmission electron microscopy (TEM). To do so, a drop of each LPHN was deposited on a carbon-coated copper grid (400 mesh). Dried samples on the grid were imaged at 120kv using TEM (JEM-2100Plus, JEOL Ltd. Japan).

#### 2.3.2. Transferrin Conjugation Efficiency

The amount of Tf conjugated to the LPHN was quantified using the Lowry assay [15], as previously described [16].

#### 2.3.3. Particle Size and Zeta-Potential Measurement

The hydrodynamic size and zeta potential of the LPHN were evaluated by photon correlation spectroscopy and laser Doppler electrophoresis techniques using the Zetasizer Pro instrument (Malvern Panalytical, UK). The LPHN (10 *µ*L) was mixed with deionized water (990 *µ*L) before being transferred into a folded capillary cell for measurement.

#### 2.3.4. Drug Entrapment Efficiency

The amount of *α*-mangostin entrapped in the LPHN was quantified using a UV-visible spectrophotometer. Briefly, nanoparticles (10 *µ*L) were mixed with methanol (990 *µ*L) and sonicated for 15 min, followed by centrifugation at 10,000 rpm at 25°C for 10 min. The optical density of *α*-mangostin in the supernatant was measured at a wavelength of 317 nm. The amount of *α*-mangostin was calculated by correlating absorbance with the standard curve of *α*-mangostin. The results were expressed as a percentage of entrapment efficiency (% EE = amount of *α*-mangostin in sample/initial amount of *α*-mangostin × 100).

#### 2.3.5. Drug Release Study

The membrane dialysis technique was used to evaluate the *in vitro* release of *α*-mangostin from the LPHN. Briefly, *α*-mangostin either formulated as Tf-LPHN, Ctrl-LPHN, or free in solution (equivalent to 400 *μ*g of *α*-mangostin in 1 mL of phosphate buffer saline (PBS) containing 0.5% Tween 20, pH 7.4) was placed into a SnakeSkin® dialysis tube (molecular weight cut-off of 3.5 kDa). The samples were dialyzed against 200 mL of phosphate buffer saline containing 0.5% Tween 20 (pH 7.4) and incubated at 37°C with continuous stirring (150 rpm). The release medium (1 mL) was collected in triplicates at specific time intervals (30 min, 1 h, 2 h, 3 h, 4 h, 5 h, 6 h, 8 h, 10 h, 12 h, 24 h, 48 h, and 72 h) and replaced with an equal volume of fresh medium. The amount of *α*-mangostin was quantified by a UV-visible spectrophotometer and reported as a percentage cumulative drug release.

### 2.4. *In vitro* Biological Characterization

#### 2.4.1. Cell Culture

MCF-7 human breast adenocarcinoma, A549 human lung adenocarcinoma, and B16–F10 murine melanoma cell lines were grown as monolayer cultures in either a MEM (for MCF-7 cells) or in RPMI-1640 medium (for A549 and B16–F10 cells) supplemented with 10% (v/v) FBS and 0.5% (v/v) penicillin-streptomycin. Cells were cultured at 37°C in an incubator with a humid atmosphere of 5% CO_2_ and subcultured every three to four days.

#### 2.4.2. Cellular Uptake Study

In order to measure the ability of the LPHN for cell internalization, the amount of *α*-mangostin accumulated within the cells was quantified using high-performance liquid chromatography (HPLC). Briefly, MCF-7, A549, and B16–F10 cells were seeded at a density of 2 × 10^5^ cells/well in 6-well plates and grown at 37°C at 5% CO_2_ for 72 hours. They were then treated with *α*-mangostin (equivalent to 10 *µ*g of *α*-mangostin in 1 mL of culture medium), either as Tf-LPHN, Ctrl-LPHN, or free in solution. After 4 hours treatment, the cells were washed twice with cold PBS (3 mL), followed by incubation with TrypLE® Express (1 mL) for 10 min to detach the cells. The cell pellets were then collected, centrifuged at 2,000 rpm for 10 min, and washed twice with cold PBS (1 mL). After that, the cell pellets were lysed with 5% Triton-X (1 mL) and incubated overnight at 37°C. The supernatant was then separated from the cell lysates by centrifugation at 10,000 rpm for 15 min and analyzed using HPLC as follows, column: ZORBAX Eclipse XDB-C18 (4.6 × 150 mm, 5 *µ*m), mobile phase: methanol and water (95 : 5) with isocratic elution for 20 min, flow rate: 0.8 mL/min, injection volume: 50 *μ*L, detection wavelength: 317 nm. The cellular uptake was calculated and reported as a percentage of cellular accumulation of *α*-mangostin.

#### 2.4.3. *In vitro* Antiproliferative Study

The MTT assay was used to evaluate the antiproliferative activity of *α*-mangostin either as Tf-LPHN, Ctrl-LPHN, blank LPHN, or a free drug solution. To do so, MCF-7, A549, and B16–F10 cells were seeded into 96-well microplates at a density of 3 × 10^3^ cells/well and grown for 24 hours in an atmosphere of 37°C, 5% CO_2_ to allow for cell attachment. After that, the medium was then removed and replaced with fresh medium containing test samples (200 *μ*L) at various concentrations (ranging from 0.3125–20 *µ*g/mL). After 48 hours of treatment, the supernatant (100 *µ*L/well) was removed and MTT solution (20 *µ*L, 5 mg/mL in PBS) was added to each well, followed by incubation in an atmosphere of 37°C, 5% CO_2_ for 2 hours. After incubation, the medium was removed, and DMSO (100 *μ*L) was then added to dissolve the formazan production in the cells. The optical density of the formazan solution was measured using a microplate reader at a wavelength of 570 nm. The results were calculated as the percentage cell viability compared with the untreated cells and fitted to obtain the IC_50_ values.

### 2.5. Statistical Analysis

All experiments were done in triplicate and the results were expressed as mean ± standard error of mean (SEM). Statistical significance was assessed by one-way analysis of variance analysis (ANOVA) and the Tukey multiple comparison test using commercial statistical software. Differences were considered statistically significant for *p* values lower than 0.05.

## 3. Results

### 3.1. Preparation and Characterization of Transferrin-Conjugated Lipid–Polymer Hybrid Nanoparticles Entrapping *α*-Mangostin

**Figure 1:**
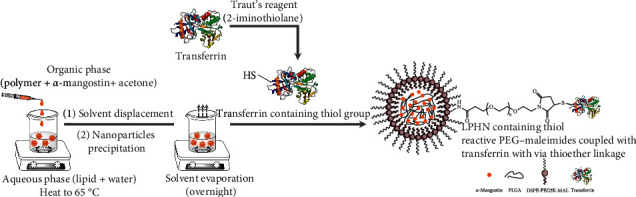
Preparation of *α*-mangostin-loaded transferrin-conjugated lipid-polymer hybrid nanoparticles.

**Figure 2:**
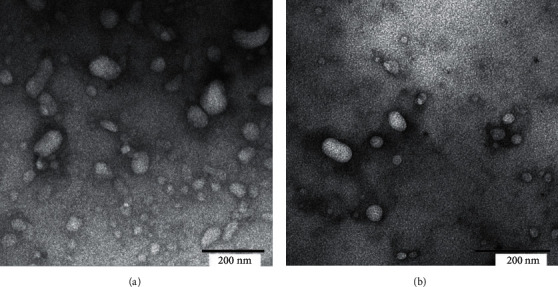
Transmission electron microscope images of Tf-LPHN (a) and Ctrl-LPHN (b) loaded with *α*-mangostin (Bar: 200 nm).

Tf-bearing and Ctrl-LPHN entrapping *α*-mangostin were successfully prepared using the one-step nanoprecipitation method (Figure 1), where the PLGA polymer (in water-miscible organic solvent) and the aqueous DSPE-PEG2K-MAL lipid dispersion were mixed and self-assembled to form spherical to ovoidal shape particles, as confirmed by TEM pictures (Figure 2). As shown in Table 1, the entrapment efficiency of *α*-mangostin within these nanoparticles was, respectively, 77.99 ± 1.15% for Tf-LPHN and 84.55 ± 0.62% for Ctrl-LPHN. The amount of Tf conjugated to the LPHN was 6.8 ± 0.1 mg (68.4 ± 0.7% of the initial Tf added). The average size of Tf-LPHN was also increased after being conjugated with Tf (294.4 ± 0.7 nm, polydispersity: 0.397 ± 0.010) compared to that of Ctrl-LPHN (128.3 ± 1.1 nm, polydispersity: 0.118 ± 0.010). Furthermore, the presence of Tf on the surface of nanoparticles significantly increased the net surface charge of Tf-LPHN (−43.7 ± 0.6 mV) in comparison with Ctrl-LPHN (−51.7 ± 0.2 mV).

### 3.2. Drug Release from the Nanoparticles

**Figure 3:**
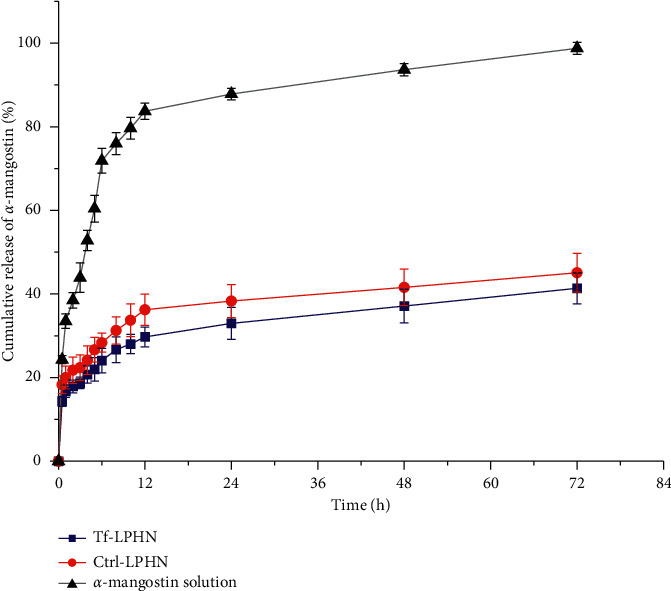
Drug release profile of *α*-mangostin formulated as Tf-LPHN and Ctrl-LPHN or as a free drug in phosphate buffer saline containing 0.5% Tween 20 at pH 7.4 over 72 hours (*n* = 3).

Both Tf-LPHN and Ctrl-LPHN exhibited a similar release profile of *α*-mangostin at pH 7.4 with initial burst release (about 16–20%) in the first hour, followed by a sustained release of *α*-mangostin over 72 hours (Figure 3). By contrast, *α*-mangostin in solution rapidly diffused through the dialysis membrane with a huge release to nearly 90% in 24 hours. The conjugation of Tf to the surface of the LPHN also had an impact on the release profile of *α*-mangostin. More precisely, *α*-mangostin was initially burst released from Tf-LPHN with a cumulative drug release of 14.3 ± 0.5% at 1 hour. Then, a steady release of *α*-mangostin from Tf-LPHN was observed with a cumulative drug release of 41.4 ± 1.5% at 72 hours. The release of *α*-mangostin from the Ctrl-LPHN followed a similar trend, but slightly faster compared to the Tf-LPHN with a cumulative drug release of 45.1 ± 1.9% after 72 hours.

### 3.3. Cellular Uptake of *α*-Mangostin

**Figure 4:**
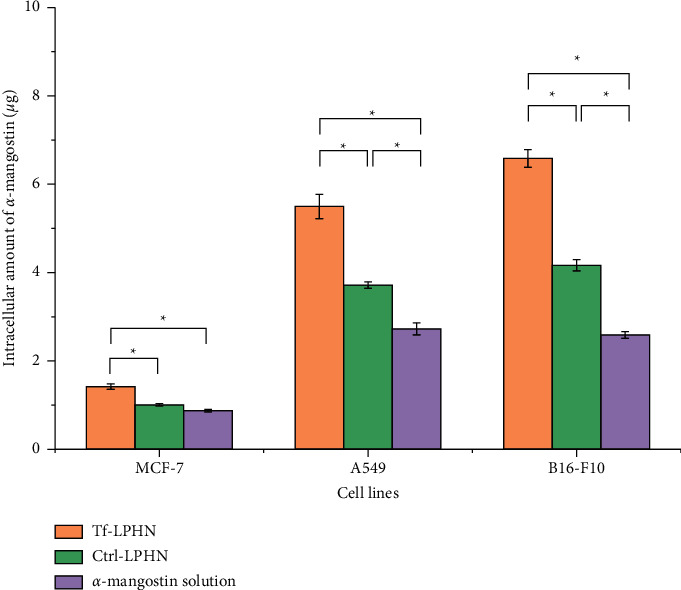
Cellular uptake of *α*-mangostin (10 *µ*g/well) either formulated as Tf-LPHN (orange) and Ctrl-LPHN (green) or as drug solution (purple), in MCF-7, A549, and B16–F10 cell lines (*n* = 6) (^*∗*^*p* < 0.05).

The intracellular accumulation of *α*-mangostin either formulated as Tf-LPHN and Ctrl-LPHN or free in solution was investigated in the three tested cell lines (Figure 4). As expected, the entrapment of *α*-mangostin in Tf-LPHN significantly increased *α*-mangostin uptake by the cells in comparison with Ctrl-LPHN and drug solution. In MCF-7 cells, the amount of *α*-mangostin accumulated in the cells treated with Tf-LPHN was 1.4-fold and 1.6-fold higher than that of Ctrl-LPHN and free drug (1.42 ± 0.06 *µ*g for Tf-LPHN, 1.01 ± 0.03 *µ*g for Ctrl-LPHN and 0.88 ± 0.03 *µ*g for *α*-mangostin solution), respectively. In A549 cells, it was 1.5-fold and 2.0-fold for Tf-LPHN in comparison with Ctrl-LPHN and *α*-mangostin solution (5.50 ± 0.28 *µ*g for Tf-LPHN, 3.72 ± 0.07 *µ*g for Ctrl-LPHN, and 2.73 ± 0.13 *µ*g for *α*-mangostin solution), respectively. The highest intracellular accumulation of *α*-mangostin was found in B16–F10 cells treated with Tf- LPHN which was significantly higher than that observed after being incubated with Ctrl-LPHN and a free drug, respectively, by 1.6-fold and 2.5-fold (6.58 ± 0.20 *µ*g for Tf-LPHN, 4.16 ± 0.13 *µ*g for Ctrl-LPHN and 2.59 ± 0.08 *µ*g for *α*-mangostin solution).

### 3.4. *In vitro* Antiproliferative Activity

**Figure 5:**
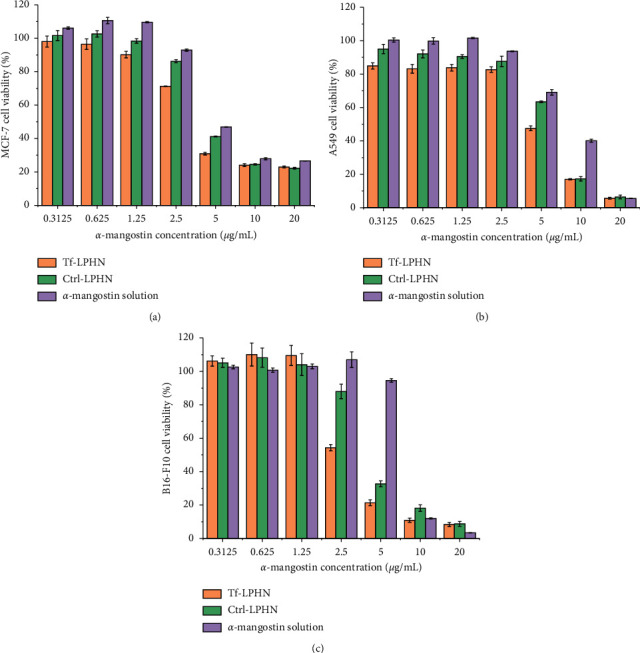
Percentage cell viability after being treated with *α*-mangostin either entrapped in Tf-LPHN (orange), Ctrl-LPHN (green), or as a drug solution (purple) on MCF-7 (a), A549 (b), and B16–F10 (c) cell lines, for 48 h (*n* = 4).

The loading of *α*-mangostin in Ctrl-LPHN led to a significant increase in its *in vitro* antiproliferative activity in comparison with that of the free solution, by at least 1.3-fold (Table 2, Figure 5). The antiproliferative efficacy of *α*-mangostin was further improved when formulating as Tf-LPHN, by 1.4-fold for MCF-7 cells, 1.7-fold for A549 cells, and 2.5-fold for B16–F10 cells, compared to that of *α*-mangostin solution following 48 hours' treatment. These results were in line with the improvement of cellular uptake following treatment with both Tf-bearing and Ctrl-LPHN. Among all the three tested cell lines, *α*-mangostin entrapped in Tf-LPHN exerted the highest antiproliferative efficacy against B16–F10 cells with IC_50_ of 2.96 ± 0.13 *µ*g/mL (equivalent to 7.21 *µ*M), while the IC_50_ in A549 cells was observed to be at 4.54 ± 0.18 *µ*g/mL (equivalent to 11.06 *µ*M). However, Tf-LPHN entrapping *α*-mangostin indicated only limited antiproliferative activity in MCF-7 cells (IC_50_: 4.16 ± 0.04 *µ*g/mL; 10.21 *µ*M), probably because MCF-7 cells are more resistant to *α*-mangostin than the two other cell lines. By contrast, we could not determine the IC_50_ of the blank LPHN in any tested cell lines, demonstrating the safety of LPHN under the tested experimental conditions.

## 4. Discussion

The promising efficacy of *α*-mangostin for cancer treatment is hindered by a lack of tumor specificity and rapid elimination *in vivo*. Therefore, we developed a Tf-conjugated LPHN that could enhance tumor-selective delivery and the therapeutic efficacy of *α*-mangostin.

The LPHN were easily prepared using the one-step nanoprecipitation method by mixing the PLGA polymer in water-miscible organic solvent (acetone) with the aqueous DSPE-PEG2K-MAL lipid dispersion in which they self-assembled. This technique is preferred (more efficient, low cost, and requires less energy and time) over the two-step method, where polymeric nanoparticles and lipid vesicles are separately prepared before being combined using ultrasonication and homogenization [17].

The thiol–maleimide ‘click' reaction was used to conjugate the LPHN with Tf [18]. This thiol-based bioconjugation technique is one of the most widely used methods for grafting delivery systems with peptides, proteins, or antibodies due to its high selectivity, rapid reaction (without catalyst), and compatibility with the aqueous conditions [19, 20]. In this study, we obtained the level of transferrin conjugation at 68.4 ± 0.7% of the initial Tf added, which was similar to our previous conjugation rate to hybrid nanoparticles by about 72% [21]. Chen and colleagues reported slightly lower conjugation efficiency (about 54%) when prepared *α*-mangostin loaded Tf-modified liposomes using N-hydroxysuccinimide (NHS)/1-ethyl-3-(3-dimethylaminopropyl) carbodiimide (EDC) coupling reagents [12].

Entrapment efficiency is one of the important parameters in the design of drug delivery systems. This parameter relies on several factors such as the types and compositions of nanocarrier, as well as the nature of drug load [22]. Our results indicated the high entrapment efficiency of *α*-mangostin in both Tf-LPHN and Ctrl-LPHN, ranging from 78–85%. Several types of delivery systems have previously been used to entrap *α*-mangostin, such as lipid-based vesicles, solid lipid nanoparticles, and polymeric nanoparticles [1]. Our LPHN formulations have a similar entrapment efficiency of *α*-mangostin to that of what was previously reported by Chen and colleagues, who developed Tf-modified liposomes, which were able to entrap 88% of *α*-mangostin [12]. A maximum entrapment eﬃciency of 51% was found when entrapping *α*-mangostin in nanostructured lipid carriers (NLC) made from lavender essential oil and cetyl palmitate [23]. Alpha-mangostin has also been loaded with PLGA nanoparticles (PLGA, lactide: glycolide 50 : 50, MW 14000–16000) with 51% entrapment [13], lower than that of our nanocarriers.

In terms of particle size and morphology, although the conjugation of Tf to LPHN led to an increase in particle size, they still displayed the required size (ranging from 128 to 294 nm) that should theoretically allow their extravasation through the leaky vasculature (cut-off size approximately 400 nm) of most tumors via the EPR effect [24]. Besides, the shape of our LPHN was spherical (or ovoidal) that can be more rapidly internalized by the cells compared to elongated ellipsoids and worm-like particles [25, 26]. Zeta potential experiments have shown that both Tf-LPHN (−43.7 mV) were bearing a negative surface charge, slightly higher than the observed in Ctrl-LPHN (−51.7 mV). This increase in the zeta potential might be due to the shielding effect of Tf conjugated on the surface of nanoparticles, including some of the positively charged amino acids of Tf as well as ferrous iron (Fe^2+^) in the protein, which might neutralize the negative surface charges of Tf-LPHN [27]. Also, it has been reported that the nanoparticles bearing a negative surface charge would reduce the risk of opsonization with serum proteins, thus avoiding rapid clearance by the mononuclear phagocyte system (MPS) and prolonging blood circulation time [28, 29]. Furthermore, our LPHN can be classified as highly stable colloidal systems as they display a zeta potential lower than −30 mV [30].

The drug release proﬁle is another parameter of controlled release systems, which might contribute to the concentration of the drug at the targeted sites as well as its therapeutic efficacy upon administration [31]. Our experiment indicated that *α*-mangostin could be efficiently sustained released (up to 45% at 72 hours) from the LPHN with initial burst release, unlike a rapid release of free *α*-mangostin. Thus, the initial burst release observed in our LPHN formulations may attribute to the presence of PEGylated lipid, which facilitates water absorption and accelerates the diffusion of *α*-mangostin entrapped in the outer layer of the polymer core through a water-filled pore, the most common drug release mechanism of polymer-based nanoparticles [32]. In addition, it is worth mentioning that low molecular weight compounds (i.e., *α*-mangostin, MW of 410.5 g/mol) also have a high propensity for burst release due to osmotic pressure [33]. Yu and colleagues described a similar release trend of *α*-mangostin [34]. In their study, *α*-mangostin was also sustained released (24.27% over 24 hours at pH 7.4) from polymeric nanoparticles made from poly (ethylene glycol)-poly (propylene glycol)-poly (ethylene glycol) (PEG-PCL-PEG) triblock copolymer, then its cumulative release increased constantly to reach a high level of 90.47% after 96 hours. In our study, Tf-LPHN exhibited a slower release of *α*-mangostin than their Ctrl-LPHN. This may be due to the ability of Tf to conjugate to the surface of nanoparticles to reduce water absorption of the nanoparticles, thus, slowing the rate of *α*-mangostin diffusion through water-filled pores. Similar to previous studies, the release of plumbagin from Tf-targeted hybrid nanoparticles (in phosphate buffer, pH 7.4) was significantly slower than nontargeted nanoparticles [21].

Cellular uptake experiments revealed that loading *α*-mangostin into the LPHN significantly increased its accumulation within the three tested cell lines. The conjugation of Tf to LPHN further enhanced the uptake of *α*-mangostin (up to 2.5-fold) when compared to a free drug solution. This improvement might be explained by the fact that Tf-modified LPHN entrapping *α*-mangostin are taken up by clathrin-mediated endocytosis, a highly speciﬁc internalization mechanism, unlike low molecular weight drugs or hydrophobic drugs, like *α*-mangostin, are able to enter the cells by nonspecific passive diffusion [29, 35]. These findings are in accordance with our previous data, which demonstrated that the cellular uptake of plumbagin entrapped in Tf-targeted hybrid nanoparticles was improved compared with unmodified nanoparticles [21]. A similar outcome was also reported by Chen and colleagues, who revealed that the cellular uptake of Tf-bearing liposomes carrying *α*-mangostin in bEnd3 cells was increased about 1.3-fold and 4-fold in comparison with unconjugated vesicles and free drug solution, respectively [12]. Guo and colleagues also found that the cellular accumulation of doxorubicin in A549 cells was more efficient by 2.8 times following treatment with Tf-targeting lipid-coated PLGA nanoparticles compared to the control formulation [36]. Among the three tested cancer cell lines, B16–F10 cells exhibited the highest cellular uptake of *α*-mangostin after treatment with Tf-LPHN. This might be explained by the different expression levels of Tf receptors on the target cell surface [37], where B16–F10 were higher than A549 and MCF-7 cell lines.

The entrapment of *α*-mangostin within the LPHN also increased its antiproliferative activity compared with a free drug in all tested cell lines. Furthermore, the conjugation of Tf to LPHN further improved the IC_50_ values, showing approximately up to 2.5-fold increase. These findings were consistent with an increase in cellular uptake of *α*-mangostin after being treated with LPHN and were in accordance with previous reports demonstrating that the therapeutic efficacy of *α*-mangostin was improved by entrapment in drug delivery systems. For example, liposomes containing *α*-mangostin improved its antiproliferative activity on HepG2 human hepatocyte carcinoma cells with IC_50_ of 1.9 *µ*M, as compared with 4.6 *µ*M for *α*-mangostin solution [38]. In another study, the cytotoxicity of water-soluble *β*-cyclodextrin-coated *α*-mangostin polymeric nanoparticles on A549 lung cancer cells was enhanced by 2.1-fold in comparison with free *α*-mangostin [39]. Wathoni and colleagues have demonstrated that *α*-mangostin chitosan-loaded kappa carrageenan nanoparticles (IC_50_ of 4.7 *µ*g/mL) exhibited a higher antiproliferative activity on MCF-7 cells than drug solution (IC_50_ of 8.2 *µ*g/mL) [40], in line with our results.

These *in vitro* results confirmed the advantage of using drug delivery systems combined with transferrin targeting ligand, which signiﬁcantly increased the cellular accumulation and antiproliferative activity of *α*-mangostin in cancer cells overexpressing Tf receptors. However, despite these promising results, the *in vivo* experiment of Tf-LPHN entrapping *α*-mangostin is required and should be further investigated for its therapeutic efficacy, biodistribution, and toxicity.

## 5. Conclusions

We successfully developed Tf-bearing LPHN entrapping *α*-mangostin, with high percentage of drug entrapment and high Tf conjugation efficiency. The nanocarriers had small particle sizes with negative charges and also displayed a sustained drug release profiles. *In vitro* evaluation showed that the cellular accumulation of *α*-mangostin was enhanced when loaded in Tf-LPHN, compared to that of nontargeted formulations and drug solutions, resulting in an improvement in its antiproliferative activity in all the tested cell lines.

The entrapment of *α*-mangostin in Tf-bearing lipid-polymer hybrid nanoparticles is therefore a highly promising strategy for cancer treatment and should be further investigated and optimized to improve its therapeutic efficacy.

## Figures and Tables

**Table 1:** Characterization of lipid-polymer hybrid nanoparticles entrapping *α*-mangostin (*n* = 3).

Sample	Transferrin conjugation (%)	Entrapment efficiency (%)	Particle size (nm)	Polydispersity index	Zeta potential (mV)
Tf-LPHN	68.4 ± 0.7	77.99 ± 1.15	294.4 ± 0.7	0.397 ± 0.010	−43.7 ± 0.6
Ctrl-LPHN	—	84.55 ± 0.62	128.3 ± 1.1	0.118 ± 0.010	−51.7 ± 0.2

**Table 2:** Antiproliferative activity of *α*-mangostin entrapped in Tf-LPHN and Ctrl-LPHN formulations, or free in solution, expressed as IC_50_ values, in MCF-7, A549, and B16–F10 cell lines, following 48 h treatment (control: blank LPHN) (*n* = 4) (n.d.: not determined) (^*∗*^*p* < 0.05*vs*. *α*-mangostin solution).

Cell lines	*Antiproliferative activity (IC*_*50*_ *: µg/mL)*			
	Tf-LPHN	Ctrl-LPHN	*α*-Mangostin solution	Blank LPHN
MCF-7	4.16 ± 0.04^*∗*^	5.27 ± 0.05^*∗*^	5.82 ± 0.06	n.d.
A549	4.54 ± 0.18^*∗*^	5.76 ± 0.05^*∗*^	7.70 ± 0.17	n.d.
B16–F10	2.96 ± 0.13^*∗*^	4.30 ± 0.07^*∗*^	7.54 ± 0.09	n.d.

## Data Availability

The datasets used and/or analyzed during the current study are available from the corresponding author on reasonable request.
